# Comparative Efficacy Study Combination of Oral Methotrexate and Prednisolone versus Oral Methotrexate in Patients with Lichen Planopilaris

**DOI:** 10.1155/2022/3792489

**Published:** 2022-10-08

**Authors:** Farahnaz Fatemi, Farifteh Esfahanian, Ali Asilian, Fatemeh Mohaghegh, Mina Saber

**Affiliations:** Department of Dermatology, School of Medicine Skin Disease and Leishmaniasis Research Center, Isfahan University of Medical Sciences, Isfahan, Iran

## Abstract

**Background:**

Lichen planopilaris (LPP) is a rare inflammatory disorder of the scalp that causes cicatricial alopecia. No therapeutic approach has been approved for this disease due to the rare frequency. Methotrexate and corticosteroid are commonly considered second- or third-line therapy. The efficacy of a combination of methotrexate and corticosteroid has been reported in some dermatological and immunological diseases. However, the efficacy of this combination in LPP is not clear. Therefore, this study aimed to compare the impact of methotrexate alone and in combination with corticosteroid on LPP.

**Materials and Methods:**

This randomized clinical trial was performed on 28 patients who referred to the dermatology clinic affiliated with Isfahan University of Medical Sciences, Isfahan, Iran during February 2015-December 2016, and 24 of them completed the trials. Fourteen patients received 15 mg methotrexate per week alone and the other fourteen subjects received 200 mg prednisolone plus 15 mg methotrexate per week. The primary outcome was Lichen planopilaris activity index (LPPAI) score. Moreover, we evaluated photographic changes and symptoms during the study.

**Results:**

The mean of LPPAI in both groups decreased during the follow-up with a similar pattern of LPPAI changes in both groups. No statistically significant difference was found between the two intervention groups regarding the LPPAI score. We found no difference in the symptoms and photographic assessments in methotrexate and combination therapy groups during follow-up. In both groups, exclusively one adverse effect was reported.

**Conclusions:**

Our results showed that methotrexate therapy with and without corticosteroids had similar efficacy and safety.

## 1. Introduction

Lichen planopilaris (LPP) is the most well-known reason for essential immune-mediated cicatricial alopecia, first depicted by Pringle in 1985. In LPP, hair follicles are specifically annihilated by a chronic lymphocytic inflammatory process that regularly brings about irreversible scarring alopecia if not treated [1]. LPP is commonly observed at the age of 40–60 years and is more frequent in women [1–3]. LPP normally represents patchy alopecia for the most part on the scalp with perifollicular erythema and scaling as the initial signs [4–9]. Patients usually complain of itching, pain, and burning in the active phase [2, 3]. Trichoscopy helps in clinical diagnosis of LPP [10]. The most common feature of lichen planopilaris in trichoscope include perifollicular scaling, absence of follicular opening, white cicatricial areas, perifollicular erythema, milky-red areas, classic white, and blue-gray dots [11, 12]. Positive anagen pull test (characterized as the recovery of even a solitary ordinary anagen hair by delicate pulling) and telogen force test (characterized as telogen count of >10% of pulled hair) are valuable clinical indications of active disease [13, 14].

The analysis depends on both clinical and histopathological discoveries. LPP has three clinical variations of a classic form, frontal fibrosing alopecia (FFA), and Graham-Little-Piccardi-Lasseur syndrome (GLPLS) [2, 15]. The occurrence of this variation has expanded significantly over the last decade [13, 16, 17]. The course of LPP is generally unpredictable, and presently accessible treatments do not prompt hair regrowth [18]. Hair regrowth is only expected when the treatment is begun at the starting points of the disease [9]. In this way, holding disease's progression is the realistic treatment goal of managing LPP [18, 19].

Various medications have been applied to treat LPP, both topically, such as intralesional corticosteroids, and systematically, namely hydroxychloroquine [20, 21]. Immunosuppressive agents, including mycophenolate mofetil and azathioprine, have been administered for more severe patients [14, 22, 23]. Methotrexate, with or without topical corticosteroids, has been utilized to treat oral [24], vulvovaginal [25, 26], anogenital, and generalized lichen planus [27, 28] with worthy clinical viability. The current study aimed to evaluate the efficacy of oral methotrexate and oral prednisolone versus oral methotrexate alone in LPP cases.

## 2. Materials and Methods

### 2.1. Patients

This randomized clinical trial was completed during February 2015-December 2016. The present investigation was conducted in the Dermatology Clinic affiliated with Isfahan University of Medical Sciences, Isfahan, Iran. This research was approved by the Institutional Review Board and Ethics Committee of the university and was registered in the Iranian Registry of Clinical Trials (IRCT2015062822959N1). The inclusion criteria entailed being diagnosed with active refractory LPP and being at the age of 18 years or older. The diagnosis was made based on clinical assessment and was confirmed by histopathological findings. Refractory LPP is defined as no response to treatment with topical medications in the past three months or systematic agents in the past six months. The exclusion criteria were pregnancy, breastfeeding, vision problems/retinopathy, psoriasis, anemia (Hb < 9 mg/dl), leukopenia (white blood cells<4000/dl), thrombocytopenia (platelets<100,000/dl), and increased liver enzymes more than two times of the upper normal limit. Another exclusion criterion was being affected by comorbidities, including diabetes mellitus, hypothyroidism, hyperthyroidism, severe hypertension, heart failure, active infection, nephropathy, and positive viral hepatic markers. Furthermore, the withdrawal criteria encompassed not showing up for follow-up visits, not taking the medication according to the study protocol, receiving other topical or immunosuppressive agents during the research, and nontolerable side effects. Written informed consent was obtained from all the participants.

### 2.2. Study Setting

All participants were randomly divided into two groups of [1] methotrexate and prednisolone and [2] methotrexate. We assigned a random number to each patient by Microsoft Excel function. Next, even and odd numbers were allocated to methotrexate and prednisolone and methotrexate groups, respectively. Complete physical examination, such as ophthalmologic examination was carried out. In addition, laboratory evaluations, including complete blood count, renal function tests, liver function tests, viral markers, and the measurement of the baseline activity of glucose-6-phosphate dehydrogenase were performed for all assented patients. Medication side effects were followed up by physical examinations and periodic laboratory evaluations, such as complete blood count, renal function tests, and liver function tests. The patients were instructed to record/report any experienced side effects.

### 2.3. Intervention

All medications were discontinued at least one month preintervention. Patients were randomly allocated to receive either 15 mg methotrexate per week alone or 200 mg prednisolone once a week plus 15 mg methotrexate per week for six months.

## 3. Outcome Measurement

The primary outcome in this study was Lichen planopilaris activity index (LPPAI) score. LPPAI was measured preintervention and 2, 4, and 6 months postintervention. LPPAI was calculated as (itch + pain + burn)/3  + (scalp erythema + perifollicular erythema + perifollicular scale)/3 + 2.5 (pull test) + 1.5 (spreading/2). Moreover, follicular keratosis was evaluated at baseline, as well as 2, 4, and 6 months after the start of the study.

Photographs were assessed at baseline and at the end of the study by two dermatologists who were blind to group allocation. The most active point was tattooed and surrounded by an ink mark in a circle of 0.5 inch radius. A standardized seven-point scale was used to interpret the photographs. The scales included −3, −2, −1, and 0 for great, moderate, slight, and no change, respectively. There was also a “do not know” option which denoted that the photographs were not suitable for proper clinical evaluation [29]. Medication side effects were carefully monitored to evaluate the safety of administered medications.

Furthermore, we assessed the impact of the intervention on the symptoms of disease, including pain (no, mild, moderate, severe), pruritus (no, mild, moderate, severe), soreness (no, mild, moderate, severe), erythema (no, mild, moderate, severe), perifollicular erythema (no, mild, moderate, severe), perifollicular scaling (no, mild, moderate, severe), pull test (no, presence), spreading (no, intermediate, presence), and follicular keratosis (no, mild, moderate, severe). These variables were scored by patients and clinicians in clinical examination.

### 3.1. Statistical Analysis

We summarized descriptive data as mean ± standard deviation (SD) for continuous variables or frequency (%) for categorical variables. Independent *t*-test and Man-Whitney *U* test were conducted to compare interval demographical and clinical variables between two groups. In addition, the Chi-square and Fisher's exact test were used for the analysis of categorical variables. Repeated measures analysis of variance (ANOVA) was applied to compare outcome variables pre- and post-intervention in each group. We also used two-level linear regression to compare the mean of changes in LPPAI between the two groups adjusting for baseline LPPAI and time. All the data were analyzed utilizing the SPSS version 24 (IBM, Armonk, NY, USA). *P*-value<0.05 was considered significant.

## 4. Results

### 4.1. Participants

**Figure 1:**
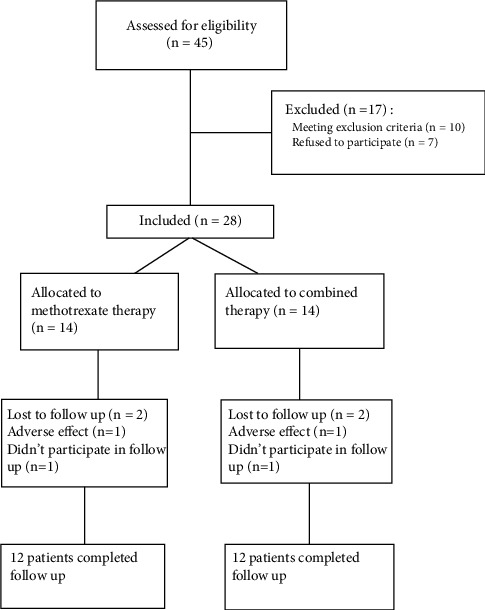
The study flowchart.

We approached 45 patients, seven of whom refused to participate and ten individuals were excluded (one patient was pregnant, two had vision problems, two had leucopenia, one had anemia, three reported comorbidities, and one had abnormal liver enzymes). Overall, 28 patients underwent randomization, 14 of whom received methotrexate and 14 received combination therapy. In both groups, 12 participants completed the study. One patient in each group left the study due to treatment adverse effects. One case in combination therapy reported uncontrolled hypoglycemia and one person in methotrexate reported anemia. The other two participants decided to leave the investigation. The flowchart of the study is shown in Figure 1.

#### 4.1.1. Baseline Features

The mean age in combination and methotrexate groups was 41.29 ± 2.34 and 47.21 ± 3.8 years, respectively. In each of the two groups, nine (64.2%) cases were female. The mean disease duration in the combination therapy and methotrexate group was 4.43 ± 0.8 months and 7.07 ± 1.34 months, respectively. We also assessed previous therapeutic experiences. All patients in both groups had used topical corticosteroid. Nine (64.2%) cases in the combination therapy group and seven (50%) people in the methotrexate group had received hydroxychloroquine. In both groups, one patient reported Hashimoto's disease. The most common initial involvement in both study groups was parietal with the frequency of 78.5% in both groups.  Table 1 demonstrates the detailed baseline categorical variables separately in both groups.

There was no significant difference between the study groups regarding demographic characteristics. The differences in clinical features between the study groups are listed in Table 2.

### 4.2. Lichen Planopilaris Activity Index

The baseline mean of LPPAI in the combination and methotrexate groups was 4 ± 2.04 and 4.9 ± 1.51, respectively. This difference was not statistically significant (*P*=0.21).  Table 3 demonstrates the mean of LPPAI at the baseline and during the study.

**Figure 2:**
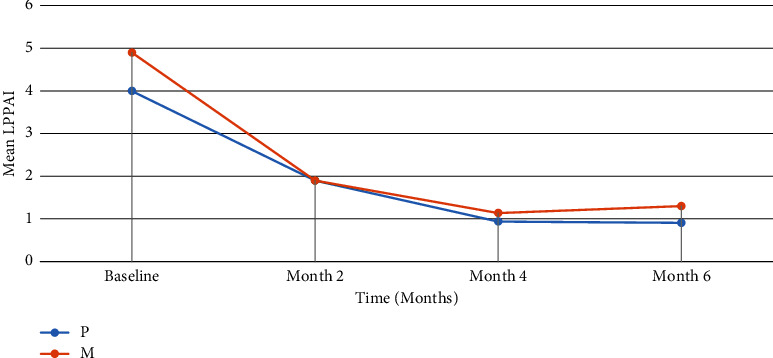
The pattern of decrease in mean Lichen planopilaris activity index in two groups.

The mean of LPPAI in both groups decreased during the follow-up. After two months, the decline in mean LPPAI was more prominent in the methotrexate group than in combination therapy. At this point, the mean of LPPAI was similar in both groups (1.9 ± 1.02 in the combination group and 1.9 ± 0.95 in the methotrexate group). After four months, the mean LPPAI in the methotrexate group was lower than that in combination therapy. A similar result was also found after six months. What stands out in Figure 2 is the similar pattern of changes in LPPAI in both groups.

This figure shows very similar pattern of change in mean Lichen planopilaris activity index in intervention and control groups.

We used two-level linear regression analysis to compare the difference between the study groups (Table 4). No statistically significant difference was found between the two intervention groups (*β* = 0.125, 95% CI:−0.439, 0.638, *P*=0.664). This analysis showed that the pattern of changes over time was statistically significant as one unit increase in time caused a 0.193 reduction in LPPAI (*β* = −0.193, 95% CI:-0.276, −0.109, *P* < 0.001). Moreover, baseline LPPAI was a significant predictor of LPPAI (*β* = 0.239, 95% CI: 0.071, 1.861, *P*=0.005).

#### 4.2.1. Photographic Assessment

Table 5 shows the comparison of photographic data. In the combination group, all patients had changes in the photographic assessment, including slight, moderate, and considerable alterations in two (16.6%), four (33.35%), and six (50%) patients, respectively. In the methotrexate group, one patient had no change in the photographic assessment, while seven (37.58.3%) represented slight, two (16.6%) showed moderate, and two (16.6%) had considerable changes. We found no statistically significant difference between combination therapy and methotrexate regarding photographic assessment.

## 5. Symptoms

We compared symptoms of diseases, including pain, pruritus, soreness, erythema, perifollicular erythema, perifollicular scaling, pull test, spreading, and follicular keratosis between combination therapy and methotrexate groups. At the baseline, spreading was more common in the methotrexate group, compared to the combination therapy group (*P* < 0.005). After two months, the frequency of perifollicular erythema was significantly different between the two groups (*P* = 0.043). We found no difference in terms of other symptoms in the methotrexate and combination therapy groups during follow-up.  Table 6 summarizes the details of the data.

### 5.1. Safety

During follow-up, two patients reported adverse effects. One participant in the methotrexate group presented severe anemia. In the combination therapy group, one individual reported uncontrolled hypoglycemia. Both patients reported adverse effects within the first two years and were excluded from the study due to these side effects.

## 6. Discussion

In the current study, we assessed the efficacy of oral methotrexate alone and in combination with oral prednisolone. The most obvious finding was the steady decrease in the LPPAI in both groups. The latter result shows that the combination of methotrexate with corticosteroid can be effective in the treatment of LPP. It is noteworthy that we found a similar pattern of changes in the LPPAI in both study groups. Furthermore, it was observed that the symptoms of study groups were not different at the baseline and throughout the investigation. We did not find a remarkable difference in photographic changes between methotrexate and combination therapy.

LPP is a rare cutaneous disorder with unknown pathogenesis. One of the main issues about LPP is the lack of a gold standard therapeutic approach. Potential treatment strategies have been suggested for LPP based on case reports and case series studies. Randomized controlled studies for guiding the treatment of LPP are scarce, which is not unexpected because of the low prevalence of the disease. We compared the efficacy of methotrexate alone with a combination with oral prednisolone. Our study provided additional support for the efficacy of methotrexate on LPP. Our results indicated a significant decline in the mean of LPPAI in patients who received methotrexate alone. Moreover, the present research provided evidence for the impact of corticosteroid combination with methotrexate. We found no difference in the mean LPPAI of the methotrexate and combination treatment groups throughout the study suggesting no superiority for either strategy in terms of efficacy.

Methotrexate is an antilymphocytic agent and has been utilized in several T-cell-mediated diseases in the past decade. Previous investigations indicated the efficacy of methotrexate on LPP [30, 31]. We previously showed that methotrexate was more effective than hydroxychloroquine in the treatment of refractory LPP [31]. Six months after the commencement of treatment, the mean of LPPAI in the methotrexate group was lower than the hydroxychloroquine group (1.51 ± 0.91vs. 3.3 ± 2.09, respectively, *P*=0.01). In addition, we found substantial improvements in the severity and frequency of symptoms in the methotrexate group [31]. In another study we showed both cyclosporine and methotrexate are effective in treating refractory Lichen planopilaris, and we proposed methotrexate as possible earlier choice [32]. This study was similar to our study. These results were in agreement with those obtained by Bulbul Baskan and Yazici. They reported clinical improvement in spectrophotometric intracutaneous analysis in the methotrexate group after three months of therapy [30]. A recent systematic review on this topic suggested methotrexate as second-line therapy in patients with LPP [33]. The mechanisms of the effect of methotrexate on LPP are still unclear. It has been shown that methotrexate declines the de novo synthesis of purines and pyrimidines through inhibiting dihydrofolate reductase. These nucleotide molecules are basic substrates for DNA synthesis in T-cell proliferation [34]. Methotrexate might modify the path of disease.

Regarding corticosteroids, high-potency topical corticosteroids and intralesional corticosteroids have been administered with successful outcomes [2,35]. This type of corticosteroid is considered a first-line treatment for limited forms of LPP [36]. In patients with predominantly inflammatory symptoms, short treatment with corticosteroids can be effective. Several case reports and case series studies showed a high success rate for treatment with systematic corticosteroids. On the other hand, these patients represented a high risk for relapse, as almost 80% of the patients experienced a relapse during the first year after drug withdrawal [4, 6, 36, 37]. Corticosteroids can be more useful in combination with other systemic therapies. Methotrexate treatment has been suggested to be effective after initial therapy with corticosteroids [20]. In the second month, the mean of LPPAI was almost similar in both groups. After two and four months, the mean of LPPAI in combination therapy was lower than that in the methotrexate group. However, these differences were not statistically significant. During the study, the frequency of symptoms was not different. Furthermore, photographic changes were not different between the two groups. A possible explanation for this might be the efficacy of corticosteroids in patients with predominantly inflammatory signs. However, the disease symptoms were similar at the beginning between the study groups, except for the spreading, which was more frequent in the methotrexate group.

Regarding safety, one patient in each group presented adverse effects, which shows that both groups experienced almost the same adverse effects. Comparison of the findings with those of other studies confirms the high tolerability of methotrexate and corticosteroids. In our previous study, one (6.7%) patient who received methotrexate experienced adverse effects (elevated liver enzymes) [31]. In an observational study, no adverse effect was reported in the methotrexate group [30].

Our research had some limitations. The major limitation of this study was the small sample size, which does not allow the generalizability of our results. Moreover, the study was limited due to the lack of information on relapse rates in patients who received corticosteroids. Despite these limitations, to the best of our knowledge, this study is the first randomized clinical trial that assessed the efficacy of the combination of oral prednisolone with methotrexate.

The current study aimed to evaluate the efficacy of methotrexate alone and in combination with corticosteroids. Our study indicated that the combination of methotrexate with corticosteroids and methotrexate alone are effective in the treatment of LPP. Moreover, our findings showed similar efficacy in both groups, and none of them were superior to the other one. Therefore, further studies are required on the current topic in order to validate our results.

## 7. Conclusions

Our results showed that methotrexate therapy with and without corticosteroids had similar efficacy and does not require the addition of oral corticosteroid. Further multi-center studies with large sample sizes and longer follow-ups are warranted.

## Figures and Tables

**Table 1:** Characteristics of participants by intervention groups at baseline

Characteristics	Combination therapy	Methotrexate	*P* Value
Age, *y*, mean ± SD	41.29 (2.34)	47.21 (3.80)	0.19
Female/male, *n* (%)	9 (64.2%)	9 (64.2%)	1.00
Duration of disease, *M*, mean ± SD	4.43 (0.84)	7.07 (1.34)	0.10
Other autoimmune disease	1 (7.14)	1 (7.14)	1.00
Positive family history	2 (14.2)	2 (14.2)	1.00
Previous treatment			
Topical corticosteroids, *n* (%)	14 (100)	14 (100)	1.00
Systemic corticosteroids, *n* (%)	4 (28.5)	3 (21.4)	0.663
Hydroxychloroquine, *n* (%)	7 (50)	9 (64.2)	0.445
Mycophenolate mofetil, *n* (%)	1 (7.14)	4 (28.5)	0.139
Cyclosporine, *n* (%)	1 (7.14)	7 (50)	**0.012**
Methotrexate, *n* (%)	1 (7.14)	5 (35.7)	0.065
Systemic retinoids, *n* (%)	0	2 (14.2)	0.142
Pioglitazone, *n* (%)	0	1 (7.14)	0.309

**Table 2:** Categorical background characteristics of participants by intervention groups.

	Methotrexate *N* = 14	Combined therapy *N* = 14	*P* Value
Cutaneous involvement	3 (21.4)	2 (14.2)	0.622
Telangiectasia	4 (28.5)	3 (21.4)	0.663
Frontal fibrosing alopecia	1 (7.14)	2 (14.2)	0.541
Facial papules	0	1 (7.14)	0.309
Mucosal involvement	1 (7.14)	1 (7.14)	1.00
Nail involvement	0	0	1.00
Eyebrow involvement	1 (7.14)	0	0.309
Follicular lichen planus	0	0	1.00
Pruritus, mean ± SD	1.07 (0.29)	2.00 (0.30)	0.49
Pain, mean ± SD	0.57 (0.20)	0.36 (0.17)	0.42
Soreness, mean ± SD	0.71 (0.27)	0.50 (0.17)	0.50
zPFE, mean ± SD	1.93 (0.27)	1.71 (0.17)	0.54
PFS, mean ± SD	1.29 (0.29)	1.36 (0.25)	0.85
Pull test, mean ± SD	0.36 (0.13)	0.43 (0.14)	0.71
Spreading, mean ± SD	1.7 (0.1)	2.0 (0.0)	0.17
Erythema, mean ± SD	1.64 (0.29)	1.07 (0.27)	0.15
FK, mean ± SD	1.86 (0.25)	1.93 (0.20)	0.82
PFP, mean ± SD	0.50 (0.20)	0.71 (0.27)	0.52

Initial involvement			
Parietal	11 (78.5)	11 (78.5)	1.00
Frontal	0	2 (14.2)	0.142
Temporal	0	1 (7.14)	0.309
Occipital	1 (7.14)	1 (7.14)	1.00
Mixed	2 (14.2)	2 (14.2)	1.00

PFE, perifollicular erythema; PFS, perifollicular scaling; FK, follicular keratosis.

**Table 3:** The change in mean Lichen planopilaris activity index over time in two groups.

Intervention Group	Baseline	Month 2	Month 4	Month 6
Combination				
*n*	14	12	12	12
Mean	4	1.9	0.94	0.91
SD	2.04	1.02	0.73	0.68

Methotrexate				
*n*	14	12	12	12
Mean	4.9	1.9	1.14	1.3
SD	1.51	0.95	1.1	0.92

**Table 4:** Two-level linear regression model analysis results of Lichen planopilaris activity index.

Characteristics	Regression coefficients	Standard error	95% CI	*P*-value
Intervention control trial	0.125	0.288	−0.439, 0.638	0.664
Time	−0.193	0.042	−0.276, −0.109	<0.001
Baseline LPPAI	0.239	0.085	0.071, 1.861	0.005

**Table 5:** The change in photographic assessment at the end of follow-up in two groups.

	No change	Slight change	Moderate change	Great change	*P* Value
Combination	0 (0.0%)	2 (16.67%)	4 (33.33%)	6 (50.0%)	0.092
Methotrexate	1 (4.17%)	9 (37.50%)	6 (25.00%)	8 (33.33%)	

**Table 6:** Comparison of sign and symptoms between combined and methotrexate therapy at the baseline and within the follow-up.

	Baseline			Two months after intervention			Four months after intervention			Six months after intervention		
	MTX	COM	*P*	MTX	COM	*P*	MTX	COM	*P*	MTX	COM	*P*
Pain, *n* (%)												
No	10 (71.43)	8 (57.14)	0.745	11 (91.67)	11 (91.67)	0.999	11 (91.67)	12 (100)	—	12 (100.0)	12 (100.0)	—
Mild	3 (21.43)	4 (28.57)		0 (0)	1 (8.33)		1 (8.33)	0 (0)		0 (0)	0 (0)	
Moderate	1 (7.14)	2 (14.29)		1 (8.33)	0 (0)		0 (0)	0 (0)		0 (0)	0 (0)	
Severe	0 (0)	0 (0)		0(0)	0 (0)		0 (0)	0 (0)		0 (0)	0 (0)	
Pruritus, *n* (%)												
No	2 (14.29)	2 (14.29)	0.877	4 (33.33)	6 (50)	0.663	5 (41.67)	10 (83.33)	0.115	6 (50)	10 (83.33)	0.244
Mild	2 (14.29)	4 (28.57)		5 (41.67)	5 (41.67)		5 (41.67)	2 (16.67)		4 (33.33)	2 (16.67)	
Moderate	4 (28.57)	4 (28.57)		3 (25)	1 (8.33)		1 (8.33)	0 (0)		2 (16.67)	0 (0)	
Severe	6 (42.86)	4 (28.57)		0 (0)	0 (0)		1 (8.33)	0 (0)		0 (0)	0 (0)	
Soreness, *n* (%)												
No	8 (57.14)	8 (57.4)	0.745	10 (83.33)	9 (75.00)	0.50	9 (75.00)	12 (100.00)	0.217	11 (91.67)	12 (100)	0.50
Mild	5 (35.71)	3 (21.43)		2 (16.67)	3 (25.0)		2 (16.67)	0 (0)		1 (8.33)	0 (0)	
Moderate	1 (7.14)	2 (14.29)		0 (0)	0 (0)		1 (8.33)	0 (0)		0 (0)	0 (0)	
Severe	0 (0)	1 (7.14)		0 (0)	0 (0)		0 (0)	0 (0)		0 (0)	0 (0)	
Erythema, *n* (%)												
No	5 (35.71)	3 (21.43)	0.580	6 (50)	4 (33.33)	0.236	6 (50)	7 (58.33)	0.999	10 (83.33)	10 (83.33)	0.999
Mild	4 (28.57)	2 (14.29)		5 (41.67)	3 (25)		5 (41.67)	4 (33.33)		0 (0)	1 (8.33)	
Moderate	4 (28.57)	6 (42.86)		1 (8.33)	5 (41.67)		1 (8.33)	1 (8.33)		2 (16.67)	1 (8.33)	
Severe	1 (7.14)	3 (21.43)		0 (0)	0 (0)		0 (0)	0 (0)		0 (0)	0 (0)	
Perifollicular erythema, *n* (%)												
No	1 (7.14)	0 (0)	0.707	1 (8.33)	2 (16.67)	0.043	7 (58.33)	7 (58.33)	0.418	5 (41.67)	7 (58.33)	0.684
Mild	5 (35.71)	6 (42.86)		10 (83.33)	4 (33.33)		5 (41.67)	3 (25)		6 (50)	5 (41.67)	
Moderate	5 (35.71)	3 (21.43)		0 (0)	4 (33.33)		0 (0)	2 (16.67)		1 (8.33)	0 (0)	
Severe	3 (21.43)	5 (35.71)		1 (8.33)	2 (16.67)		0 (0)	0 (0)		0 (0)	0 (0)	
Perifollicular scaling, *n* (%)												
No	2 (14.29)	4 (28.57)	0.766	2 (16.67)	3 (25)	0.067	5 (41.67)	3 (25)	0.528	2 (16.67)	3 (25)	0.564
Mild	7 (50)	4 (28.57)		7 (58.33)	2 (16.67)		4 (33.33)	2 (16.67)		5 (41.67)	2 (16.67)	
Moderate	3 (21.43)	4 (28.57)		1 (8.33)	6 (50)		2 (16.67)	4 (33.33)		4 (33.33)	4 (33.33)	
Severe	2 (14.29)	2 (14.29)		2 (16.67)	1 (8.33)		1 (8.33)	3 (25)		1 (8.33)	3 (25)	
Pull test, *n* (%)												
No	8 (57.14)	9 (64.29)	0.50	12 (100)	12 (100)	—	12 (100)	12 (100)	—	12 (100)	12 (100)	—
Presence	6 (42.86)	5 (35.71)		0 (0)	0 (0)		0 (0)	0 (0)		0 (0)	0 (0)	
Spreading, *n* (%)												
No	0 (0)	8 (57.14)	**<0.001**	8 (66.67)	9 (75)	0.202	9 (75)	12 (100)	0.217	10 (83.33)	12 (100)	0.478
Intermediate	1 (7.14)	3 (21.43)		1 (8.33)	3 (25)		1 (8.33)	0 (0)		2 (16.67)	0 (0)	
Presence	13 (92.86)	3 (21.43)		3 (25)	0 (0)		2 (16.67)	0 (0)		0 (0)	0 (0)	
Follicular keratosis, *n* (%)												
No	0 (0)	0 (0)	0.162	0 (0)	0 (0)	0.648	0 (0)	0 (0)	0.158	1 (8.33)	1 (8.33)	0.784
Mild	4 (28.57)	7 (50)		8 (66.67)	6 (50)		8 (66.67)	11 (91.67)		8 (66.67)	10 (83.33)	
Moderate	7 (50)	2 (14.29)		3 (25)	3 (25)		4 (33.33)	1 (8.33)		3 (25)	1 (8.33)	
Severe	3 (21.43)	5 (35.71)		1 (8.33)	3 (25)		0 (0)	0 (0)		0 (0)	0 (0)	

MTX: methotrexate; COM: combined therapy.

## Data Availability

The data are available from the first author upon request.
